# Using Inundation Extents to Predict Microbial Contamination in Private Wells after Flooding Events

**DOI:** 10.1021/acs.est.3c09375

**Published:** 2024-03-13

**Authors:** Kyla R. Drewry, C. Nathan Jones, Wesley Hayes, R. Edward Beighley, Qi Wang, Jacob Hochard, Wilson Mize, Jon Fowlkes, Chris Goforth, Kelsey J. Pieper

**Affiliations:** † Department of Civil and Environmental Engineering, Northeastern University, Boston, Massachusetts 02115, United States; ‡ Department of Biological Sciences, University of Alabama, Tuscaloosa, Alabama 35401, United States; § Haub School of Environment and Natural Resources, University of Wyoming, Laramie, Wyoming 82072, United States; ∥ Division of Public Health, North Carolina Department of Health and Human Services, Raleigh, North Carolina 27609, United States; ⊥ State Laboratory of Public Health, North Carolina Department of Health and Human Services, Raleigh, North Carolina 27609, United States

**Keywords:** private wells, well water, inundation mapping, microbial contamination, flood boundaries

## Abstract

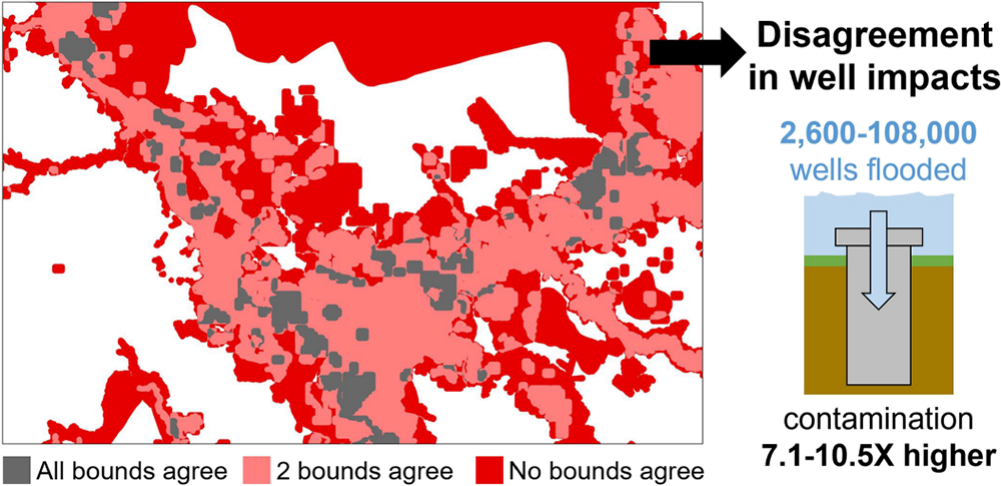

Disaster recovery poses unique challenges for residents reliant on private wells. Flooding events are drivers of microbial contamination in well water, but the relationship observed between flooding and contamination varies substantially. Here, we investigate the performance of different flood boundaries—the FEMA 100 year flood hazard boundary, height above nearest drainage-derived inundation extents, and satellite-derived extents from the Dartmouth Flood Observatory—in their ability to identify well water contamination following Hurricane Florence. Using these flood boundaries, we estimated about 2600 wells to 108,400 private wells may have been inundated—over 2 orders of magnitude difference based on boundary used. Using state-generated routine and post-Florence testing data, we observed that microbial contamination rates were 7.1–10.5 times higher within the three flood boundaries compared to routine conditions. However, the ability of the flood boundaries to identify contaminated samples varied spatially depending on the type of flooding (e.g., riverine, overbank, coastal). While participation in testing increased after Florence, rates were overall still low. With <1% of wells tested, there is a critical need for enhanced well water testing efforts. This work provides an understanding of the strengths and limitations of inundation mapping techniques, which are critical for guiding postdisaster well water response and recovery.

## Abstract 2

This study provides insight on the use of inundation mapping techniques to identify microbial contamination in private wells following flooding events, which can guide outreach and water quality recovery efforts.

## Introduction

Flooding events pose serious public health risks to communities who rely on private wells for their drinking water supplies.^1−6^ Previous studies document the connectivity between flood waters and well water (i.e., when private wells are submerged during a flood) leading to higher rates of microbial contamination and associated waterborne pathogens.^4−7^ This is problematic as consumption of contaminated well water can lead to adverse health outcomes such as gastrointestinal illness and impaired birth outcomes.^8−10^ Due to barriers in postdisaster testing (e.g., costs, knowledge or awareness, transportation),^3−5,11^ there are still limited data on the occurrence and transport of microbial contamination during flooding events. The relationship between flooding and contamination,^3,5,6^ coupled with a limited understanding of the impact on the well community,^12−16^ emphasizes the need for outreach strategies targeting well users in flooded areas.

Well water sampling efforts after disasters have traditionally focused on flood-impacted areas, but there are no standardized methods used to identify flood-impacted private wells. A variety of techniques have been used to map the boundary of the flood including inundation extents derived from combinations of hydraulic mapping, terrain-based modeling, and remotely sensed data.^17−20^ Using existing flood hazard maps^6^ and satellite-based products,^5^ studies have documented the relationship between flooding and microbial contamination in well water. However, the strength of the association observed varied substantially—some found only a weak association between contamination and flooding,^3,6^ while others observed strong associations.^5^ In addition, relationships developed using inundation extents were not as strong as those of user-reported flooding. After Hurricane Harvey, microbial contamination was 8 times higher in flooded versus nonflooded wells based on user-reported data compared to only 2–3 times higher using inundation extents.^5^ Inundation extents may not fully capture flooding risks for the well community due to techniques and limitations used to develop the boundaries (e.g., rainfall-derived, coastal storm surge, overbank flooding).^21−24^ Further, there is no knowledge of how differences in inundation extents impact the assessment of well water impacts (e.g., contamination and damage) after a flooding event.

In September 2018, Hurricane Florence made landfall on Wrightsville Beach, North Carolina (NC) as a slow-moving Category 1 hurricane. The hurricane produced record rainfall totals and widespread flooding, which resulted in two-thirds of counties declaring a state of emergency.^25^ In response, the NC Department of Health and Human Services (DHHS) provided free well water testing to well users in affected counties immediately following the storm. In this study, we used the DHHS testing data to (1) examine the relationship of flooding and well water contamination using various inundation mapping techniques; (2) evaluate the spatial variability in ability of inundation mapping techniques to identify well water contamination inundation extents; and (3) compare participation in routine and post-Florence well testing. As each inundation extent mapping technique will produce a different boundary, understanding the strengths and limitations (e.g., data integrity, implementation time, and access)^17,20,26,27^ of the approach is critical for guiding postdisaster well water response and recovery.

## Methods

### Study Area and Data Sets

After Hurricane Florence, a federal state of emergency was declared for North Carolina. In response, the Federal Emergency Management Agency (FEMA) designated 28 counties where individuals and households were eligible to apply for financial and direct recovery services.^28^ These FEMA-designated counties served as our study area (Figure S1), which spanned 17,069 square miles (44,210 km^2^; 32% of the state) and contained an estimated 311,843 private wells.^29^

**Figure 1:**
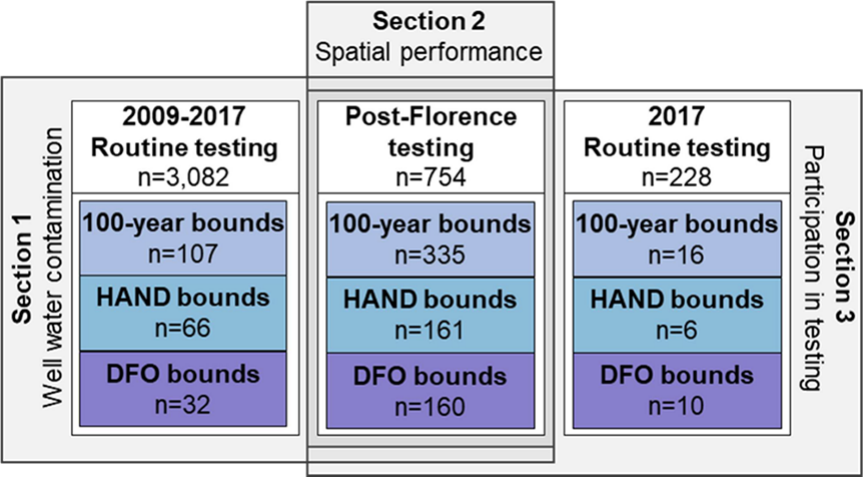
Summary of data sources and data sets used in our analysis. The data sources (gray boxes) include (1) Section 1: well water contamination rates were compared between the 2009–2017 routine and post-Florence samples within the study area and by flood boundary; (2) Section 2: the ability of flood boundaries to identify microbial contamination was evaluated spatially using post-Florence samples; and (3) Section 3: participation in routine and post-Florence DHHS testing was evaluated. All samples were collected between September and November.

We received well water testing data from the DHHS State Laboratory of Public Health (“State Lab”). Between September 2018 and August 2019, 1285 samples were submitted for free total coliform (TC; indicating surface water contamination) and *Escherichia coli* (EC; fecal contamination) analysis following Hurricane Florence (Figure 1). Local health departments requested sampling bottles from the State Lab and distributed them to interested residents upon request. Participants were recommended to collect a sample at the wellhead or outdoor tap after at least 5 min of flushing. In our analysis, we removed samples that were not collected between September and November following the storm (September 14–November 30; *n* = 194). We selected this time frame as most samples (80%) were collected and contamination rates were highest during this period (Figure S2 and Table S1). We removed samples from private wells outside our study area (*n* = 3), incomplete addresses (*n* = 8), and repeat samples (*n* = 326). In total, we included 754 samples from unique private wells (Table S2). Using ArcGIS Pro 3.1., we geocoded wells to point locations on a parcel level based on sample addresses.

Between July 2009 and June 2017, 48,879 samples were submitted for TC and EC analysis of which 12,920 samples (26.4%) were from our study area. These tests were collected under routine sampling conditions (i.e., not after a disaster), typically after the construction of new wells per the 2008 NC General Statute §87–97.^30^ The median analysis fee for microbial testing is $90 but ranged from $25 to $250 based on the local health department.^11^ As before, samples were recommended to be collected at the wellhead or outdoor spigot after flushing for at least 5 min. We removed samples that were not collected between September 1 and November 30 (*n* = 9833) and samples with incomplete addresses (*n* = 5). In total, we included 3082 samples from 2117 unique private wells. We did not remove repeat samples as prior work documents that repetitive sampling provides a more accurate contamination rate.^31^ In addition, we subset this data set to explore the 228 samples collected from unique wells between September 1 and November 30 in 2017 (i.e., the year before Hurricane Florence; Table S2). Again, we geocoded well locations from the sample addresses.

We collected 2020 US Decennial Census data on the White alone non-Latino population at the block level to determine the Black, Indigenous, and People of Color (BIPOC) percentage. From the 2020 American Community Survey, we collected block group level data on the percent of adults living below the poverty level, percent of adults renting, and percent of adults living without medical insurance.

### Methods for Predicting Inundation Extents

Without standardized approaches, studies use various inundation mapping techniques and data sources to characterize the extent of flooding.^3,5,6^ In this study, we used three inundation mapping techniques to explore differences in predicted inundation extents (herein referred to as flood boundaries). We added a 100 m buffer to all flood boundaries^5^ to prevent exclusion of wells from the sample that were located on a different part of the parcel than the geocoded point location. This buffer increased sample size but had no significant impact (test of proportions, *p* > 0.05 for all differences) on overall contamination and testing rates (Table S3). All spatial analysis was conducted in ArcGIS Pro 3.1.

The FEMA 100 year flood hazard area (“100 year”) flood boundary is derived using both hydrologic and hydraulic models to identify locations that have a 1% chance of flooding in any given year.^24^ Given the FEMA 100 year boundaries are not specific to Hurricane Florence (i.e., represents the 100 year flood occurring across the entire study area), this boundary had the largest extent (Table S4) with an estimated 4332 square miles (11,222 km^2^; 25.4% of the study area) of flooded area.

The Height Above Nearest Drainage (HAND)-derived inundation extents incorporates reach-averaged relationships between water depth and river discharge based on Manning’s equation.^17^ We obtained peak discharges from the National Water Model for Hurricane Florence for river reaches throughout the study area and used those discharges to estimate water depths and corresponding flood extents. In our study, we used depth–discharge relationships that were based on the uncalibrated HAND flood boundary, which assumes a uniform Manning’s roughness value of 0.05 for all river reaches. To account for uncertainty in the mapping approach (e.g., simulated discharges, reach-averaged hydraulic relationships, and 10 m horizontal resolution elevation data), a filter was used to refine flooding. In particular, we applied a threshold water depth of 1 m to define the flooded pixels. The HAND boundary estimated that 1878 square miles (4864 km^2^; 11.0% of the study area) was inundated (Table S4).

The Dartmouth Flood Observatory (DFO) uses remotely sensed observations collected from optical and radar sensors onboard the Landsat, Sentinel, and MODIS satellites.^32,33^ These satellite data are used to classify flooded and nonflooded pixels, which are validated by ground observations and then processed to develop flood boundaries estimating the maximum flooding extent of the storm. The DFO boundary estimated that 888 square miles (2302 km^2^; 5.2% of the study area) was inundated following Florence (Table S4).

### Statistical Analyses

#### Post-Florence Well Water Contamination

To characterize the impacts of Hurricane Florence on well water contamination rates, we compared post-Florence TC and EC positivity rates to routine conditions among all samples and among samples within the three flood boundaries. To evaluate the impact of flood boundaries and other potential drivers of increased contamination rates, we developed two logistic regression models.^34^ The response variables were the probability that the sample was TC or EC positive. The predictors for both models were categorized into four groups: (1) flooding characteristics (three variables; whether a test was inside or outside 100 year, HAND, or DFO boundaries); (2) demographic characteristics (four variables; block estimates for percent BIPOC, block group estimates for percent renters, block group estimates for percent living below the poverty level, and block group estimates for percent of block group without medical insurance); (3) well population characteristics (two variables; number of wells in each tract and percent of population using wells in each tract); and (4) testing characteristics (two variables; number of wells tested in each tract and percent of wells tested in each tract). All predictors were standardized and scaled to have a mean of zero. Regression analysis was performed in R version 4.2.3 by using the tidyverse, sf, and raster packages.

#### Spatial Variability in Ability of Flood Boundaries to Identify Contamination

To measure spatial autocorrelation, we used the Getis-Ord Gi* statistic from the Optimized Hotspot Analysis in ArcGIS Pro 3.1. We explored the clustering of microbial contamination after Hurricane Florence within our study area and identified two clusters. Contamination rates inside and outside clusters were compared using the Test of Proportions. We calculated the accuracy, sensitivity, and specificity of the flood boundaries using a contingency analysis (Table S5) within the study area, within clusters, and at the tract level. Sensitivity was the rate of correctly identifying true positives (EC-positive samples inside the flood boundary). Specificity was the rate of correctly identifying true negatives (EC negative tests outside of the flood boundary). Accuracy was the rate of correctly identifying positive and negative tests inside of each boundary. To evaluate spatial variability in the performance of the flood boundaries, we compared the tract level sensitivity of each flood boundary against the percent of the area flooded. We defined low flooding as less than 25% of the tract predicted as flooded by the inundation product and high flooding as more than 25% of the tract predicted as flooded. We defined low sensitivity as less than 50% of true positive samples detected and high sensitivity as more than 50% of true positive samples detected.

#### Participation in Routine and Post-Florence Well Water Testing

To evaluate participation in routine and post-Florence testing, we analyzed testing rates in 2017 routine and post-Florence data sets at the county level and by each flood boundary. We compared testing distributions using the Spearman’s Rank test. In addition, to evaluate representation in testing, we calculated the demographic characteristics of the well using and tested well locations in the study area at the block group level.^35^ In brief, we used a weighted average approach using block group level demographic data and weighted by either well estimates or post-Florence testing to develop average demographic estimates within the study area and each boundary.

## Results and Discussion

Well water contamination increased after Hurricane Florence, but rates varied by flood boundaries. Of the 2.5 million residents in the FEMA-designated counties, an estimated 20.7% of residents (518,000 people) were reliant on private wells for their drinking water supply. After Hurricane Florence, 41.2% of wells tested were positive for TC and 11.7% were positive for EC. These rates were 1.2 and 7.8 times higher than those under routine conditions, which were, on average, 35.0% positive for TC and 1.5% for EC (Table S2). These observed increases in contamination are similar to impacts observed after Hurricane Harvey, where TC and EC rates were 1.2 and 2.8 times higher than preflooding conditions.^5^ While data of microbial contamination after hurricanes are still limited, our results confirm that the risk of well water contamination is heightened following flooding events.

**Figure 2:**
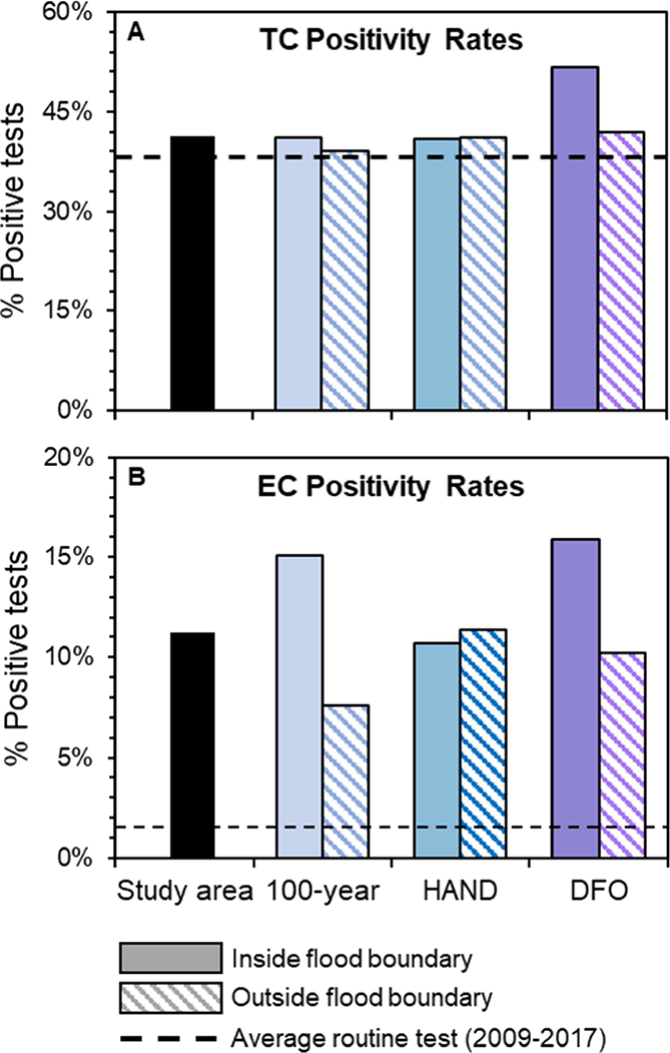
Observed (A) TC and (B) EC positivity rates in the study area and inside and outside the flood boundaries under routine conditions and after Hurricane Florence.

**Figure 3:**
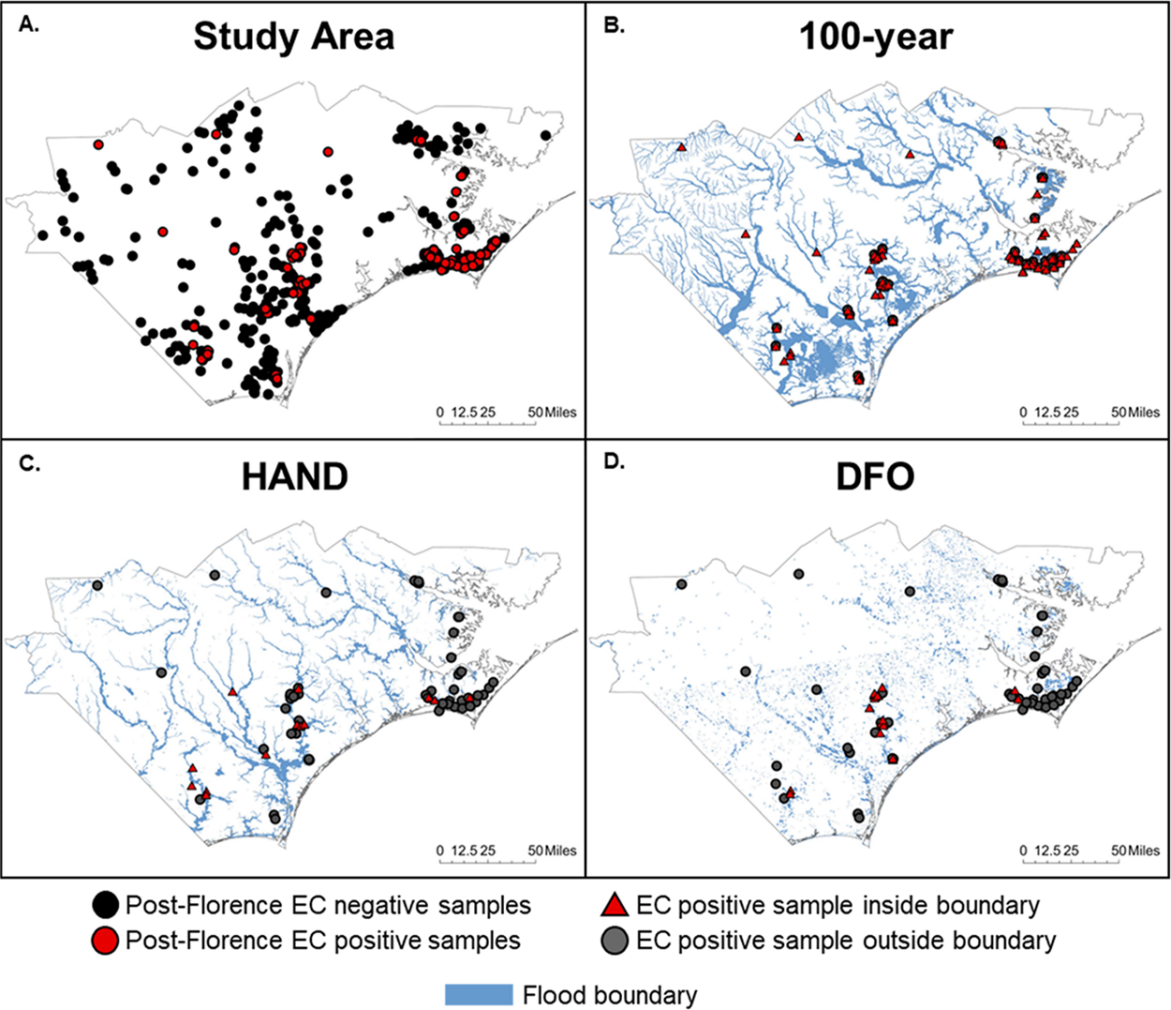
EC contaminated wells inside and outside of the three flood boundaries used in the analysis for the 3 month period following Hurricane Florence within the 28 FEMA-designated counties.

Researchers have documented that contamination rates are highest among private wells that are inundated during flooding events.^3,5^ However, the strength of the correlations observed varied based on the inundation mapping technique used (e.g., DFO modeled inundation) and/or data collected (e.g., surveying residents). When examining contamination rates within the three flood boundaries used in this study, we found that contamination rates were up to 1.2 times higher for TC and 10.9 times higher for EC among wells within flood boundaries compared to those outside flood boundaries (Table S6). However, there was substantial disagreement between observed contamination within the flood boundaries. Contamination rates varied from 40.8 to 53.1% of wells tested positive for TC and 10.5–16.3% for EC (Figure 2). Moreover, the boundaries identified different TC and EC-positive samples, and no boundary identified all positive samples within the study area (Figures 3 and S3). As expected, the area of the flood boundaries also varied substantially—the 100 year boundary was 2.9 and 7.9 times larger than the HAND and DFO boundaries, and the HAND boundary was 2.7 times larger than the DFO boundary. While all boundaries captured flooding in areas of major inundation, disagreement occurred along the fringe of the boundaries and based on type of flooding (Figure S4). In particular, 13% of the area considered as flooded by the HAND boundary and 49% of the DFO boundary was outside the 100 year boundary.

While being located within a flood boundary was a driver of contamination, we observed variation in these relationships depending on the flood boundary. In our logistic regression, we observed that private wells were 1.3 times more likely to test positive for TC in areas classified as being flooded by the DFO boundary (Figure S5 and Table S7) and 1.3 times more likely to test positive for EC in areas predicted to be flooded by the 100 year boundary (Figure S6 and Table S8). However, neither regression model had a strong predictive power. The models had limited ability to discriminate positive versus negative samples, as the models could correctly identify positive samples only 50–60% of the time and had a precision between 2 and 20% (Tables S9 and S10). Thus, the variables used in model development were not capturing all mechanisms of well water contamination, suggesting that flooding is not the sole predictor of microbial contamination and that there are additional drivers (e.g., system characteristics and user behavior). This was further supported by the presence of positive samples among wells outside the flood boundaries −35.7 to 41.3% of samples were TC positive and 8.4–12.0% were EC positive (Figure 2 and Table S6). Further, contamination rates outside the flood boundaries were up to 1.2 times higher for TC and 8.0 times higher for EC than during routine conditions (Figures 2 and 3). Of particular concern was the HAND-based boundary because it was the only boundary where contamination rates were higher outside than inside the flood boundary (41.3 vs 40.9% TC; 12.0 vs 10.5% EC, Figures 3 and S2).

The approaches used to develop the flood boundaries impacted area delineation, which influenced observed contamination rates. The 100 year boundary was the largest as the boundary did not incorporate storm-specific factors (i.e., assumes that the entire study area experienced flooding resulting from the 100 year storm). As a result, the boundary overpredicted flooded areas. However, the boundary was inclusive of different types of flooding (i.e., riverine and coastal flooding), as it was developed considering both hydrodynamic modeling of riverine and coastal processes using relatively high-resolution topographic and bathymetric data (i.e., characteristics of the underwater terrain). The HAND boundary was derived from a combination of elevation data, storm-specific discharges, and reach-averaged relationships between water depth and discharge,^17^ rendering the boundary storm-specific. However, HAND only captured overbank, riverine flooding,^22^ leading to an underprediction of flooded areas. Further, the boundary was dependent on estimated parameter values (e.g., channel roughness, assumed uniformly to be 0.05).^27^ The DFO boundary was also storm-specific, as the boundary was developed from validating satellite-based changes in water body extents with ground observations and availability of satellite observations during peak flooding conditions. The boundary was inclusive of both riverine and coastal flooding. However, it is subject to the availability of imagery at the time of flooding, and for optical sensors, cloud cover impacts water detection,^36^ resulting in a likely underprediction of flooded areas.

### Fecal Contamination Was Clustered and the Ability of Flood Boundaries to Identify the Contamination Varied Spatially

Based on the Getis-Ord Gi*, we determined that there were two clusters of TC and EC contamination (Figure S7): an inland cluster where the hurricane made landfall and a coastal cluster located along the southern portion of the NC coast. There was substantial overlap in the TC and EC clusters with 98% agreement on samples within the inland cluster and 97.5% within the coastal cluster. Here, we explore the cluster of EC-positive samples.

The inland EC cluster was composed of 7 tracts within Duplin and Pender counties, while the coastal EC cluster was 17 tracts within Carteret, Beaufort, and Pamlico counties. The clusters were small compared to our study area, with the inland cluster land area of 1609 square miles (4167 km^2^; 9.4% of study area) and coastal cluster land area of 871 square miles (2255 km^2^; 5.1% of study area). While roughly two-thirds of submitted samples (*n* = 481 of 754, 64%) came from either cluster, the clusters contained 78% (*n* = 68 of 88) of the EC-positive samples. Although not significantly higher, attributed to the smaller sample size, EC positivity rates were higher in the inland cluster (13.0 vs 11.8%, test of proportions, *p* = 0.61) and coastal cluster (14.6 vs 11.8%, *p* = 0.23) compared to the entire study population.

The sensitivity (i.e., ability to identify EC-positive samples inside flood boundary) varied substantially across the three flood boundaries; the average sensitivity of the 100 year boundary was 59.1%, HAND boundary was 19.3%, and DFO boundary was 29.5%. This resulted in low false negative rates (i.e., EC-positive samples outside flood boundary) of 4.8, 9.4, and 6.9%, respectively (Tables S10–S12). Within the inland flooding cluster, the 100 year and DFO boundary identified 62.5 and 68.8% of EC-positive samples while the HAND boundary identified 25.0% samples (Table 1). In the coastal flooding cluster, the 100-year boundary identified 66.7% EC-positive samples, while the HAND and DFO boundary identified less than 12% samples.

**Table 1:** Performance of Flood Boundaries to Identify EC-Positive Samples within Target Areas

	area of interest		100 year		HAND		DFO	
	spatial extent	*n*_EC+ total_	% EC+ inside	*n*_EC+ inside_	% EC+ inside	*n*_EC+ inside_	% EC+ inside	*n*_EC+ inside_
sensitivity	study area	88	59.1%	52	19.3%	17	29.5%	26
	inland cluster	32	62.5%	20	25.0%	8	68.8%	22
	coastal cluster	36	66.7%	24	11.1%	4	5.5%	2
	outside clusters	20	40.0%	8	25.0%	5	10.0%	2

	spatial extent	*n*_EC– total_	% EC– outside	*n*_EC– outside_	% EC– outside	*n*_EC– outside_	% EC– outside	*n*_EC– outside_
specificity	study area	666	57.5%	383	78.4%	522	79.9%	532
	inland cluster	214	50.0%	107	78.0%	167	64.0%	137
	coastal cluster	204	47.5%	97	90.2%	184	92.6%	189
	outside clusters	248	72.2%	179	79.4%	197	83.1%	206

In keeping with sensitivity, the specificity (i.e., ability to identify EC-negative samples outside flood boundary) varied substantially. The 100 year boundary had the lowest specificity (57.5%), while HAND and DFO boundaries were higher at 78.4 and 79.9%. This trend was opposite than the observed sensitivity trends. Further, the false positive rates (i.e., EC-negative samples inside flood boundary) were higher than the observed false negative rates at 37.5, 19.1, and 14.8%, respectively (Tables S10–S12). HAND had the highest specificity within the inland cluster at 78% and HAND and DFO boundaries both had specificities >90% in the coastal cluster. Outside the clusters, the DFO boundary has the highest specificity, at 83.1% (Table 1). The 100 year boundary had the lowest specificity across all target areas, attributable to the large nonstorm-specific boundary.

**Figure 4:**
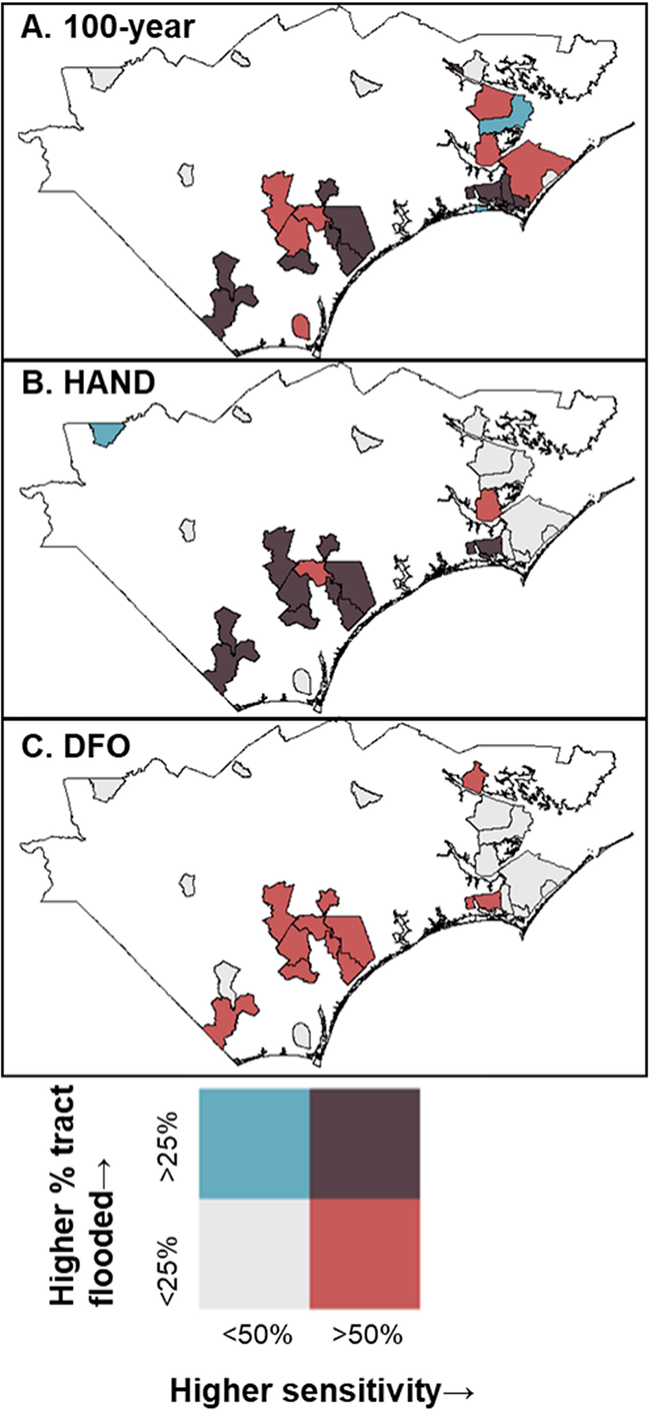
Bivariate maps reporting estimated percent of tract area flooded versus sensitivity for each flood boundary.

At the tract level, the average sensitivity and specificity improved for all of the boundaries. Sensitivity was 75% for the 100 year flood, 44.4% for HAND, and 42.8% for DFO and specificity was 73% for 100 year flood, 88% for HAND, and 90% for the DFO boundary (Table S13). When considering the percentage of flooded area within the tract, many tracts were considered to have low flooding −41.9% of tracts using the 100 year boundary, 58.1% using the HAND boundary, and 100% using the DFO boundary (Figure 4). However, the sensitivity improved among the tracts that had high flooding with an average of 89.0% among the 18 tracts within the 100 year boundary and 61.5% among the 13 tracts within the HAND boundary. Although within these highly flooded tracts, we observed lower average specificities: 67% for the 100 year boundary and 81% for the HAND boundary (Table S14). This is expected, as tracts with higher flooded areas likely included more overall samples and therefore more false positives.

Differences in boundary sensitivity and specificity were once again related to the disagreement between areas determined to be flooded. The 100 year boundary was developed considering both riverine and coastal flooding, leading to high performance in both clusters, as evidenced by high sensitivities both within the clusters and by tracts. However, as the 100 year boundary was not storm-specific, it overestimated flooded areas, as evidenced by the lower specificities. The HAND boundary captured only overbank flooding along rivers,^22^ which led to higher performance in the inland cluster and poor sensitivity across the tracts. Further, HAND had poor performance in the coastal cluster because HAND has an elevation threshold to account for uncertainty propagation,^27^ which results in underestimating flooding in low-lying areas where flooding depths are shallow. Despite variable sensitivity, HAND had high specificity both within clusters and by tract due to the use of storm-specific data. The DFO boundary had the highest sensitivity in in the inland cluster with good specificity because the boundary incorporated actual, event specific, observations to define inundation extent.^36^ The DFO inundation product may have had lower sensitivities in the coastal cluster due to the availability of imagery over this area during peak flooding or cloud cover impacts on water classification.

There were 20 EC-positive samples within 7 tracts that were not within the two clusters, which occurred in the northern and western portions of our study area. The ability of the flood boundaries to capture these EC-positive samples was low as boundaries had sensitivities less than 50%. These EC-positive samples outside the clusters highlight that flood-related contamination is not the sole driver of well water contamination. Even under routine conditions, an average of 1.5% of private wells tested EC positive with annual rates varying between 0.6 and 2.1% (Table S2). Thus, these 20 wells may have become contaminated during Hurricane Florence as nonflooding rainfall events can increase contamination rates^37^ but may be associated with other contamination mechanisms.

### Participation in State-Sponsored Well Water Testing Increased after Hurricane Florence

From September to November, the year before Hurricane Florence, routine water samples from 228 wells (0.1% of the well population) within our study area were submitted to the State Lab (Table S15). On average, 12 samples were submitted per county (range of 0–57 samples, Figure S8 and Table S16) despite well populations of 1612 to 124,606 wells (Table S17). After Hurricane Florence, 2.6 times more samples were submitted (754 samples, 0.2% of the well population) than the time period between September and November the year before the storm, with an average of 27 samples per county (ranges of 0–233 samples). Private wells within the flood boundaries were less likely to have been sampled and tested during routine conditions than wells outside the boundaries. In 2017, only 9–20 samples were submitted (0.01–0.4% of wells) within the three flood boundaries (Figure S8 and Table S15), and 160–335 samples were submitted (0.3–6.1% of wells) after Hurricane Florence. Overall, testing within the boundaries increased 16.8–17.8 times after Hurricane Florence (Figure S9).

Our findings are consistent with prior work documenting changes in perception and awareness of water quality issues after disasters and increased participation in free or low-cost testing opportunities.^3,38^ Interestingly, there was no relationship between prior participation and postdisaster testing; counties with higher testing rates under routine conditions did not have higher disaster testing (Spearman’s ρ = 0.16, Figure S8 and Table S16). To illustrate, Pender and Carteret counties had 9.3 and 16.6 times more well water samples submitted after Hurricane Florence compared to routine conditions (*n* = 203 vs *n* = 22 and *n* = 233 vs *n* = 14, respectively), while there were 7 counties (29%) where no well water samples were submitted after Hurricane Florence.

In our study area, our block group estimates suggest that 36.8% of well users identify as BIPOC and 15.2% are living below the poverty level (Figure S10 and Table S18).^35^ Understanding the flooded areas and associated well populations is critical to developing emergency response efforts. Prior studies have reported that low-income and BIPOC well users are more likely to have systems that are susceptible to contamination and subsequently more likely to have water quality issues.^39,40^ Further, not all properties that flooded may need assistance; some properties may not be occupied (e.g., vacant rentals, seasonal homes) or may have appropriate treatment installed. When exploring participation in post-Fllorence testing, we observed that estimated demographics of the testing population remained consistent across all three flood boundaries (Figure S10). Our block group estimates suggested that 26.1% of the well users that participated in testing were BIPOC and 12.8% were below poverty level. Interestingly, 17.8% of tests were estimated to be associated with rental units compared with an estimated 24.9% in our study area. Our estimates show the BIPOC and renting tested populations to be 10.7 and 7.1% less than that of the well using population. This suggests there could be an undersampling of these populations in our study area.

Participation in testing was an important factor in our regression analysis, as the results highlighted that increased testing in rural areas was associated with increased contamination. In particular, we observed private wells were 3.9 times more likely to be EC positive for every 1% increase of testing in the tract but 4.2 times less likely to be EC positive for every additional test conducted in the tract (Figures S5 and S6). This is in keeping with trends noting higher percentage using well water but smaller per capita well waters in rural settings.^11^ Similar trends were documented after Hurricane Harvey, where flooding impacted both rural and urban settings, but wellhead submersion and subsequent contamination were more common in rural settings.^5^

## Implications

### Inundation Mapping Techniques Can Enhance Disaster Response Efforts but the Flood Boundary Used Will Shape Outreach Efforts

Consistent with prior studies,^3,5^ we observed that private wells estimated as flooded were up to 7.1–10.5 times more likely to have EC contamination than estimated nonflooded private wells. Providing flood boundaries that identify well populations at-risk of flood-induced contamination has the potential to enhance recovery outreach efforts. However, due to differences in spatial extent of the boundaries, there were an estimated 2600–108,400 private wells that may have been flooded during Hurricane Florence. With an estimated 311,800 private wells in the study area, this encompasses 0.8–34.7% of the well population (Tables S4 and S17). Without understanding the magnitude of flooding, evaluating the extent of testing participation in flooded areas is not feasible, as between 0.4 and 18.9% private wells may have been tested after Hurricane Florence.

There are key uncertainties associated with community-science well sampling, rendering the use of flood boundaries to predict well water contamination challenging. Although testing increased under disaster conditions, contextualizing the impacts of the flooding is challenging, as there are limited baseline data for direct comparison. Our analysis documents the relationship between flooded areas and EC-positive samples, but drivers of EC contamination are much more complex. Contamination can be attributed to nonflooding conditions during an event (e.g., rainfall) as well as routine conditions (e.g., poor construction).^37,41−43^ Residents may mitigate contamination through treatment methods such as shock chlorination,^3,5,44^ which can reduce rates of contamination observed. There are also well-documented barriers to testing,^38^ which skews and limits the quantity of data collected. In our testing, participation by residents was voluntary, and not all health departments requested sampling bottles from the State Lab. Further, timing of sampling, access to transportation, and extent of damage are examples of other key barriers to participation in postdisaster testing.^3^ While these flood boundaries provided an enhanced understanding of the ability to use inundation mapping techniques to predict well water contamination, better input data is needed to increase validity of our comparisons. Overall, inundation mapping techniques can be used to identify flood boundaries, which will enhance outreach efforts, but caution should be used when attempting to predict individual well impacts.

### While No Boundary Identified All contamination, the 100 Year Flood Boundary Was the Most Sensitive and Readily Accessible

Although microbial contamination rates were elevated in all flood boundaries, no boundary was able to identify all of the observed contamination. Further, there were trade-offs in the accuracy, sensitivity, and specificity for each flood boundary (Tables 2 and S10–S12). This was attributed to the ability of the inundation mapping techniques to identify different types of flooding (e.g., riverine, surface, and coastal). While the 100 year floodplain incorporates all flooding mechanisms, it likely overestimated impacts, as it assumes the entire region experienced the 100 year event. The HAND and DFO boundaries likely underestimated impacts, which was attributed to challenges associated with reach-averaged hydraulics and uniform roughness when developing HAND-derived inundation^27^ and challenges associated with space-time sampling of satellites.^21^ Currently, the 100 year boundary has the highest sensitivity, which is ideal for identifying the population at-risk of microbial contamination. However, this boundary has poor specificity and the lowest accuracy due to the nonstorm-specific spatial extent, which is not optimal for distribution of limited resources. As the 100 year floodplain boundary is publicly available in most of the U.S., this data layer can be downloaded prior to flooding events (unlike HAND which needs to be generated and DFO which is available only for larger storms). Thus, the 100 year boundary provides a readily accessible and enhanced method for understanding private wells susceptible to flooding, which can be used to enhance private well disaster outreach efforts.

**Table 2:** Evaluation of Each Estimated Flood Boundary in Terms of the Validity of Contamination Detection, Accessibility, and Associated Errors^a^

flood boundary		100 year	HAND	DFO
validity	accuracy	good	good	good
	sensitivity	good	very poor	poor
	specificity	good	very good	very good
accessibility	timing	pre-existing boundary	near real-time boundary	data days to weeks after
	download	available in most of US	needs to be generated	only available for larger storms
	flood characteristics	extent	extent and depth	extent
errors		not storm-specific	reach-averaged hydraulics, uniform roughness	space-time sampling of satellites

a Very poor: 0–25%; poor: 26–50%; good: 50–75%; very good: 76–100%.

Further work is needed to increase the predictive power of storm-specific flood maps. In this study, we used an uncalibrated HAND boundary, which assumes constant hydraulic parameters, such as channel roughness. The optimization of HAND-modeled inundation maps^22,23^ to adjust roughness coefficients, and other hydraulic parameter estimations could potentially produce a storm-specific boundary with improved predictive power. This boundary can be developed in near real time, with predicted boundary values days to weeks prior to the storm. While there are well-known limitations for using satellite-derived products (e.g., cloud coverage, satellite location), the launching of the Surface Water and Ocean Topography (SWOT) satellite and enhancement of other remote sensing devices^21,45,46^ serves to improve the calibration and validation of inundation mapping techniques and availability of imagery by integrating multiple satellites.^47,48^

Overall, flooding events are continuing to occur with increasing intensity and frequency,^49,50^ and access to safe well water will continue to be of concern. Flood boundaries produced from inundation mapping techniques must be rapidly and easily used to guide testing locations following future flooding events. While the 100 year flood boundary is the best current option, the balance between performance and accessibility must be further optimized for the adoption of these techniques by health departments and other emergency response agencies.

## Supporting Information Available

Additional water testing data and figures regarding microbial contamination rates, logistic regression outputs, and additional flood boundary and demographic data are provided (PDF)
