# Supplementary material for: Using Inundation Extents to Predict Microbial Contamination in Private Wells after Flooding Events
Source: Environ Sci Technol. 2024 Mar 13;58(12):5220–8. doi: 10.1021/acs.est.3c09375 (PMC10976889; doi:10.1021/acs.est.3c09375)
Supplement: Supplementary file 1 — es3c09375_si_001.pdf [file es3c09375_si_001.pdf]

Using inundation extents to predict microbial contamination in private wells after flooding events

Kyla R. Drewry,[1] C. Nathan Jones,[2] Wesley Hayes,[1] R. Edward Beighley,[1] Qi Wang,[1] Jacob Hochard,[3] Wilson Mize,[4] Jon Fowlkes, [4] Chris Goforth,[5] Kelsey J. Pieper[1*]

[1]Department of Civil and Environmental Engineering, Northeastern University, Boston, MA, 02115, USA
[2]Department of Biological Sciences, University of Alabama, Tuscaloosa, AL, 35401, USA
[3]Haub School of Environment and Natural Resources, University of Wyoming, Laramie, WY 82072, USA
[4]Division of Public Health, North Carolina Department of Health and Human Services, Raleigh, NC 27609, USA
[5]State Laboratory of Public Health, North Carolina Department of Health and Human Services, Raleigh, NC 27609, USA

*Corresponding Author: k.pieper@northeastern.edu, 400 Snell Hall, Northeastern University, 360 Huntington Avenue, Boston, MA 02115, USA

DESCRIPTION OF SUPPORTING INFORMATION

Figure S1. FEMA designated counties
Figure S2. Testing and contamination trends in the weeks following Florence.
Figure S3. Post-Florence total coliform positive and negative samples
Figure S4. Comparison of inundation extents using the three flood boundaries.
Figure S5. Odds ratios results from total coliform regression output
Figure S6. Odds ratios results from *E. coli* regression output
Figure S7: Optimized hotspot analysis for (A) total coliform and (B) *E. coli*.
Figure S8. Comparison of 2017 routine tests to 2018 post-Florence testing by county.
Figure S9. Number of wells and testing rates within target areas
Figure S10. Demographics of the well using and tested populations
Table S1. Comparison of testing and contamination rates between post-Florence and routine testing in the weeks following Florence.
Table S2. Comparison of testing and contamination rates within the study area and by flood boundary among the post-Florence and routine testing.
Table S3. Comparison of testing and contamination rates within the study area and by flood boundary among post-Florence and routine testing with and without buffer.
Table S4. Estimated area and number of private wells potentially impacted by Hurricane Florence inside and outside flood boundaries.
Table S5. Contingency table used for validity calculations
Table S6. Well water contamination rates within the study area and by flood boundary among the three datasets.
Table S7. Coefficient results from the total coliform logistic regression
Table S8. Coefficient results from the *E. coli* logistic regression
Table S9. Model performance for total coliform and *E. coli* logistic regressions
Table S10. Contingency table for 100-year boundary identifying *E. coli* samples
Table S11. Contingency table for HAND boundary identifying *E. coli* samples
Table S12. Contingency table for DFO boundary identifying *E. coli* samples
Table S13. Percent of tract estimated to be flooded and sensitivity by flood boundary
Table S14. Tract level summary statistics of high flooded tracts of flood boundary ability to identify *E. coli* positive samples based on percent of tract flooded.
Table S15. Number of samples taken within the study area and by flood boundary among the three datasets.
Table S16. Number of samples collected during the three campaigns by county.
Table S17. County population and well using characteristics
Table S18. Average block group level demographics of the well using and tested populations

SUMMARY: 29 Pages, 10 Figures, 18 Tables

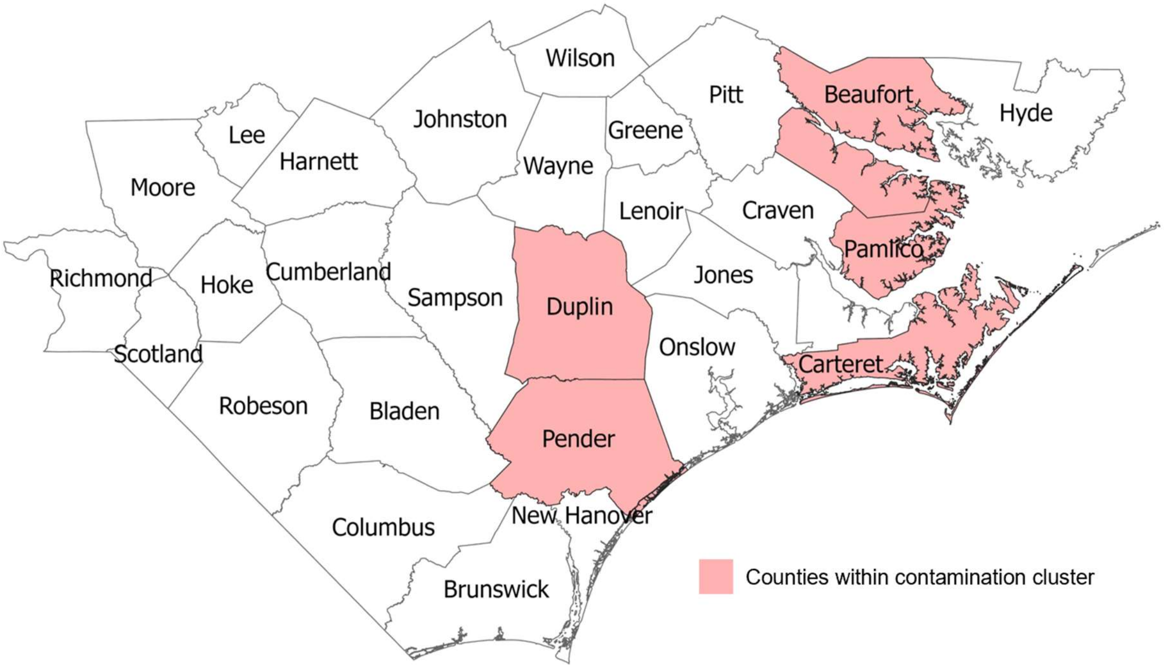

Figure S1. Federal Emergency Management Agency designated counties where individuals and households were eligible to apply for financial and direct recovery services.[34] Counties shaded in pink represent the five counties identified in the two contamination clusters.

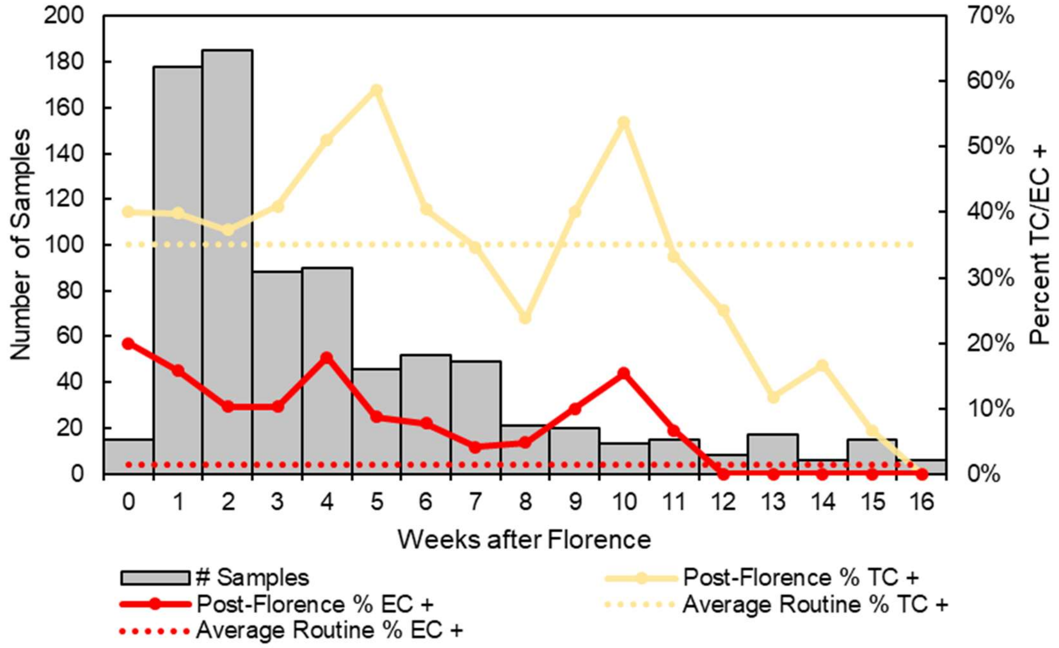

Figure S2: Number of weekly samples collected and total coliform (TC) and *E. coli* (EC) contamination rates in the 16 weeks following Hurricane Florence (September 14 – November 30) and the average TC and EC contamination rates during routine testing over the nine-year (2009-2017) testing period between September 1 – November 30.

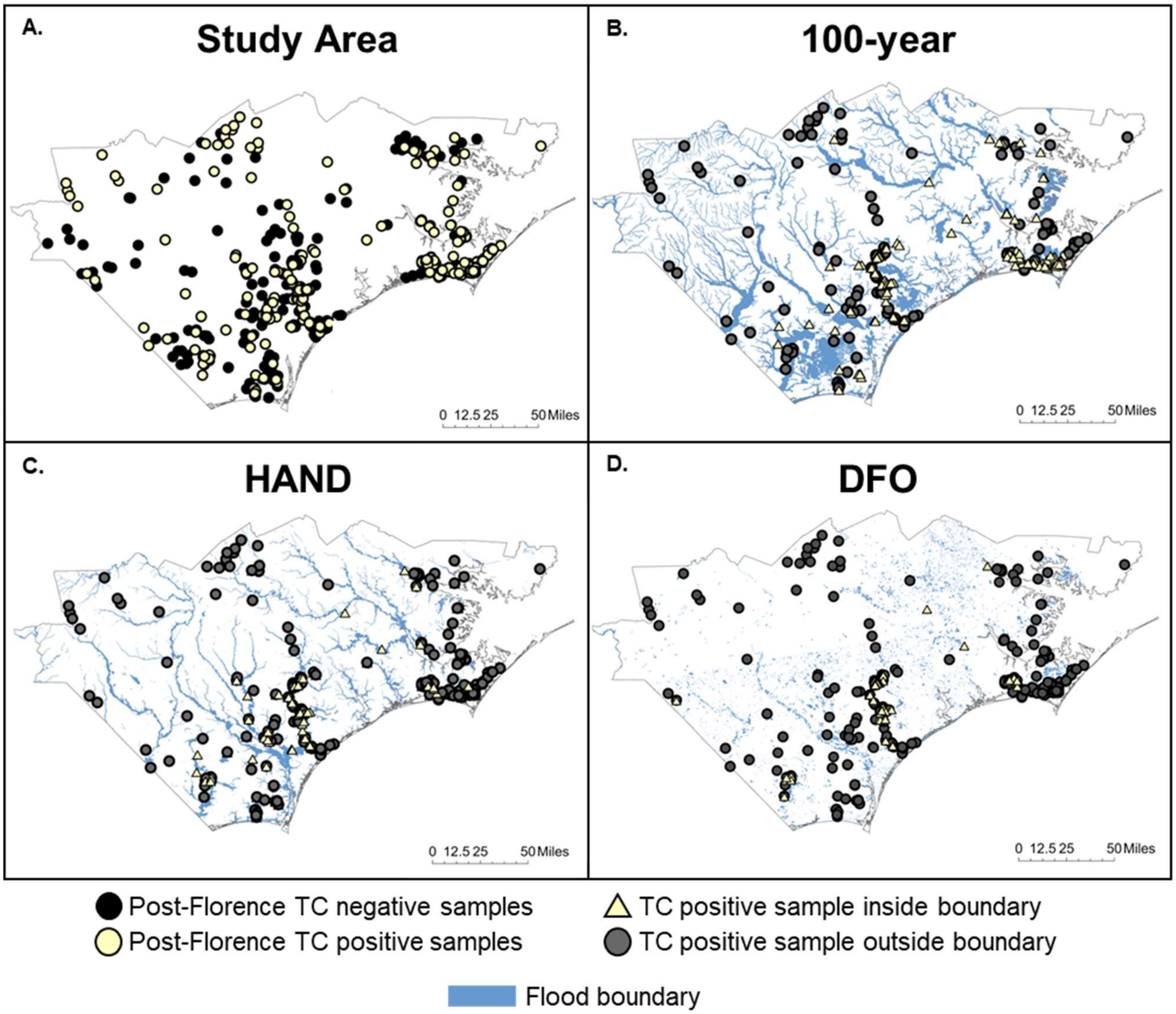

Figure S3. Post-Florence TC positive and negative samples (A) with the study area, (B) inside and outside the 100-year boundary; (C) inside and outside the HAND boundary; and (D) inside and outside the DFO boundary. Samples were collected between September and November 2018. TC: total coliform.

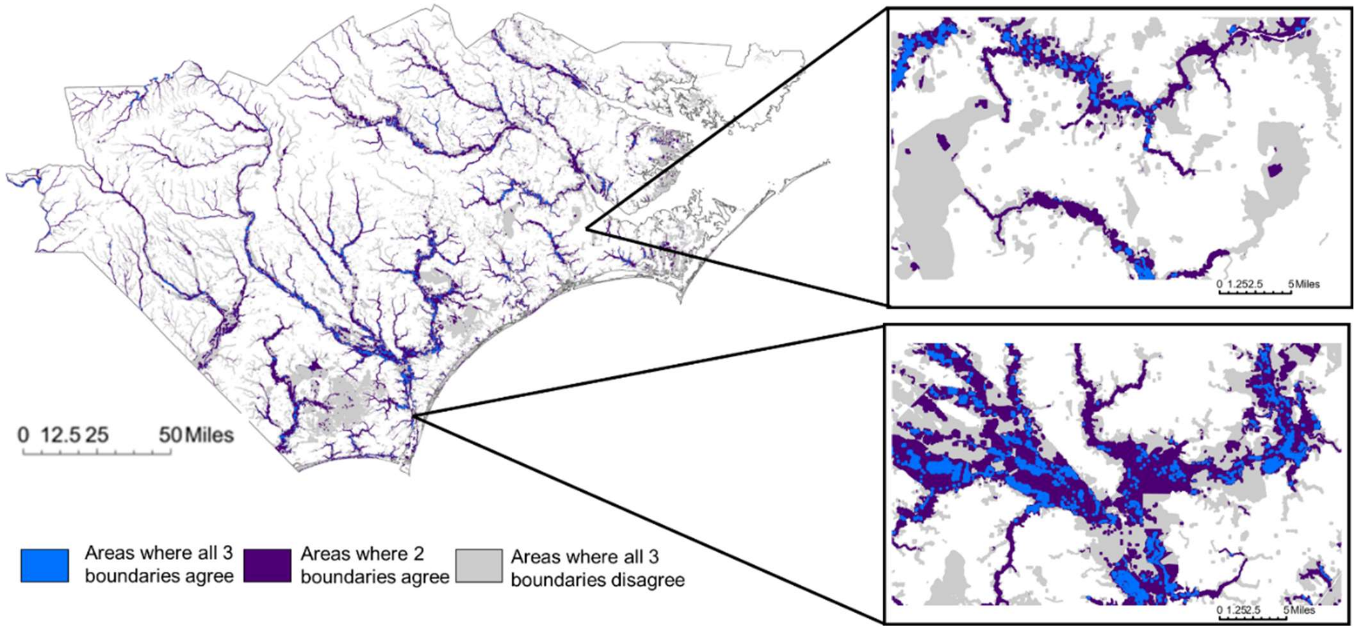

Figure S4. Comparison of inundation extents using the three flood boundaries. Blue: areas where all 3 boundaries reported there was flooding. Purple: areas where 2 of 3 boundaries reported there was flooding. Gray: areas where 1 of 3 boundaries reported there was flooding.

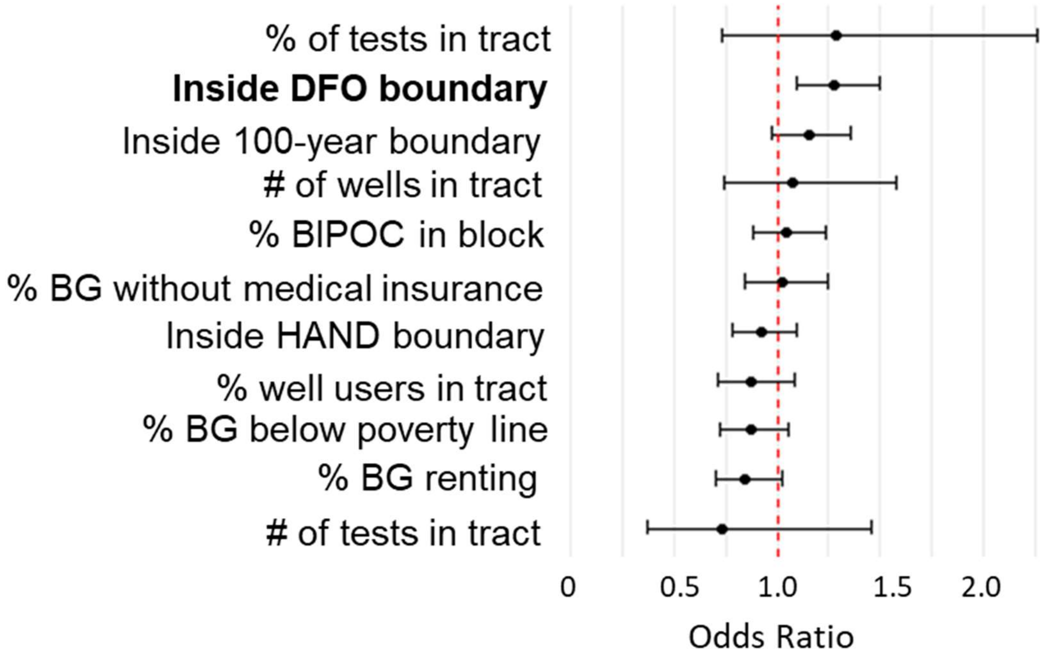

Figure S5. Odds ratios results from total coliform regression output. Bold text indicates significant variables.

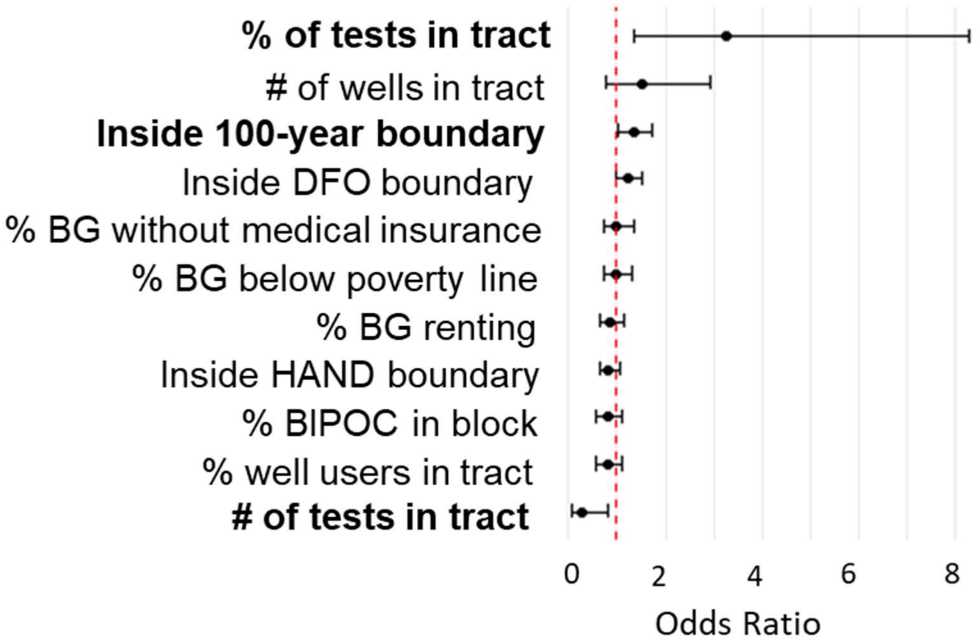

Figure S6. Odds ratios results from *E. coli* regression output. Bold text indicates significant variables.

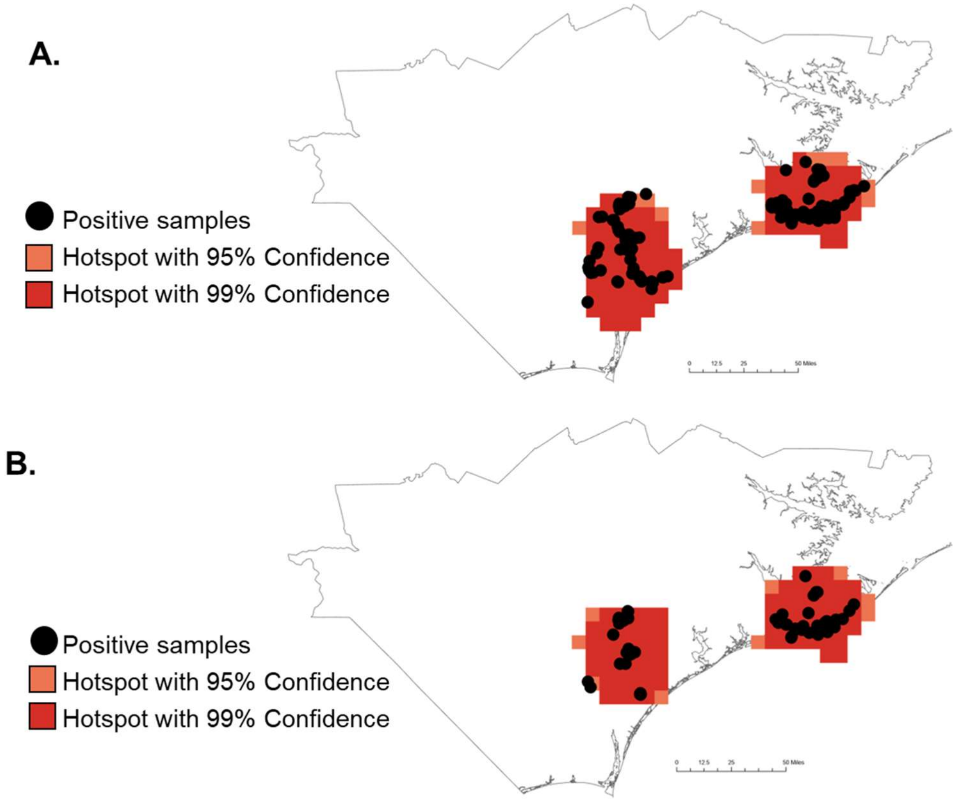

Figure S7. Optimized hotspot analysis for (A) total coliform (n=209 of 311 TC positive samples) and (B) *E. coli* (n=68 of 88 EC positive samples). The Inland Cluster (left cluster) contained 115 TC positive samples and 32 EC positive samples. The Coastal Cluster (right cluster) contained 94 TC positive samples and 36 EC positive samples.

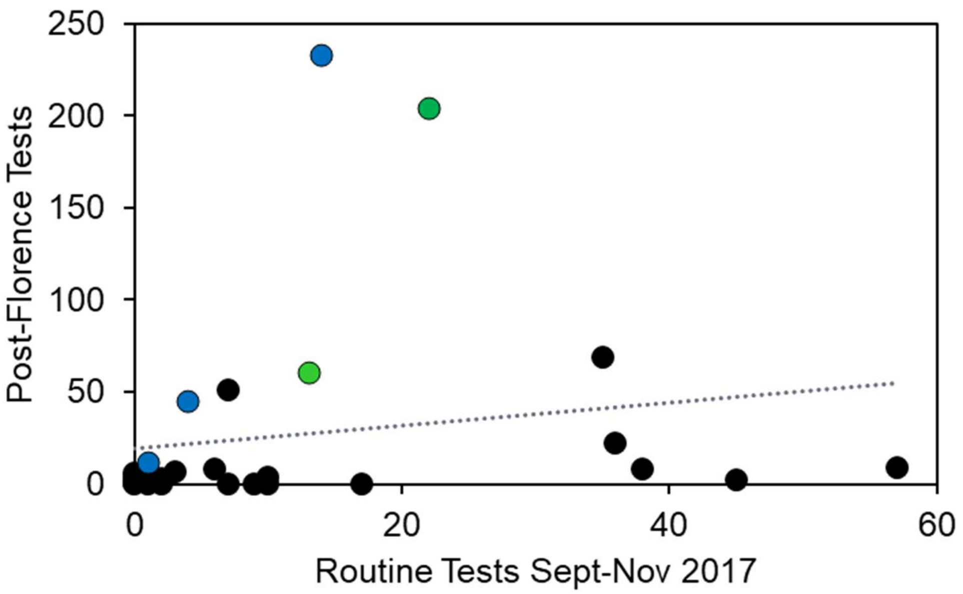

Figure S8. Comparison of 2017 routine tests to 2018 post-Florence testing by county. The dashed line represents the relationship between the datasets (ρ=0.02). Blue data represents counties within the coastal cluster; green data represents counties within the inland cluster; black data represents counties outside the identified clusters.

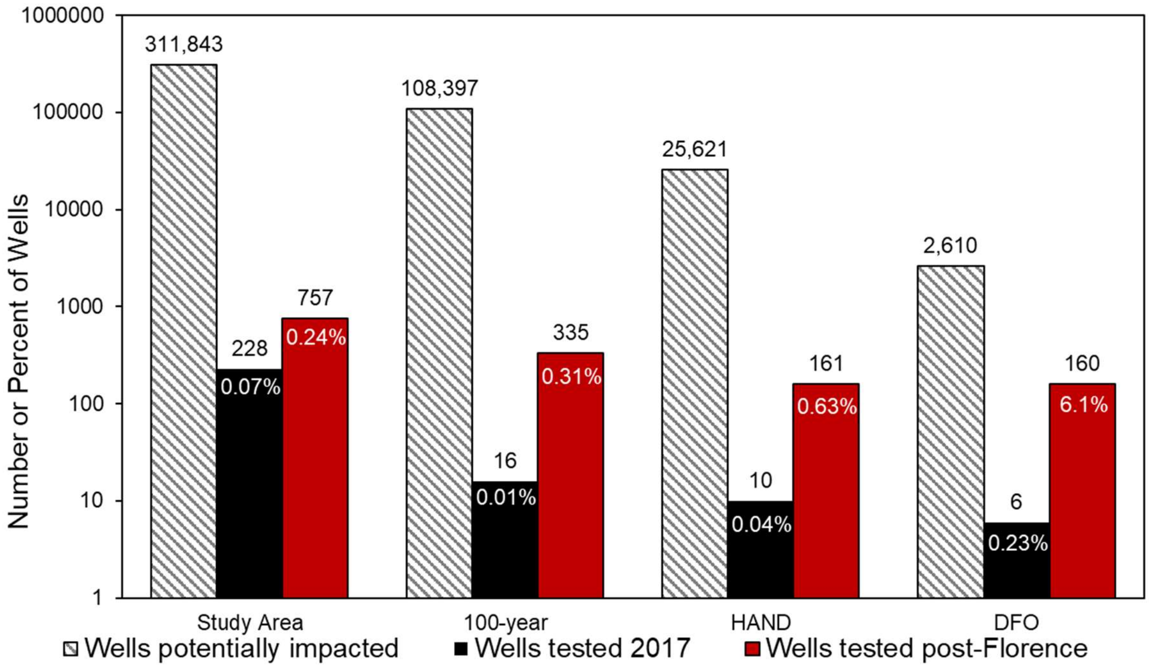

Figure S9. Number of wells and percent of wells tested within target areas among the three datasets used in this study.

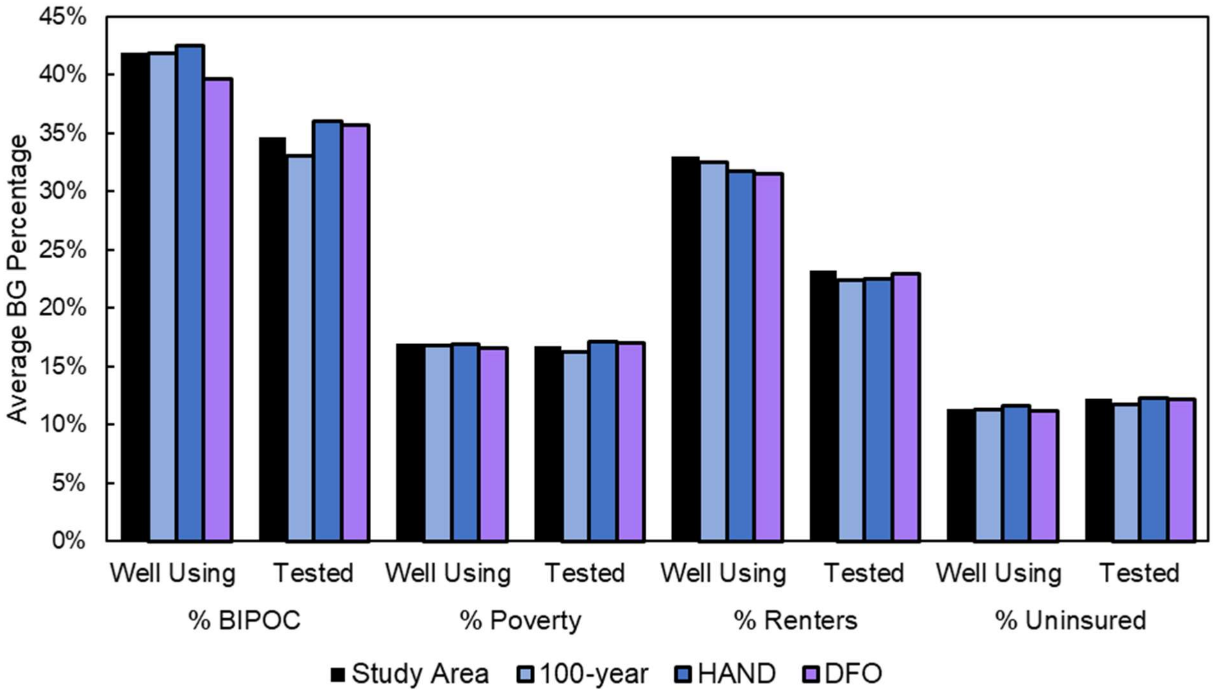

Figure S10. Average demographic estimates of the well using and tested populations after Hurricane Florence using block group level data. Black: study area; Light blue: 100-year boundary; Dark blue: Hand boundary; Light purple: DFO boundary.

Table S1. Changes in number of post-Florence samples collected, post-Florence total coliform and *E. coli* contamination rates, and routine contamination rates in the sixteen weeks following Hurricane Florence.

Weeks after Florence	# Samples	% TC positive		% EC positive	
	Post-Florence	Post-Florence	Routine	Post-Florence	Routine
0	15	40.0%	42.3%	20.0%	2.0%
1	178	39.9%	34.7%	15.7%	1.5%
2	185	37.3%	21.9%	10.3%	0.0%
3	88	40.9%	19.9%	10.2%	1.2%
4	90	51.1%	23.7%	17.8%	2.1%
5	46	58.7%	19.8%	8.7%	0.4%
6	52	40.4%	17.6%	7.7%	1.9%
7	49	34.69%	25.3%	4.1%	1.3%
8	21	23.8%	26.7%	4.8%	2.0%
9	20	40.0%	33.9%	10.0%	2.4%
10	13	53.9%	34.9%	15.4%	2.1%
11	15	33.3%	36.4%	6.7%	3.4%
12	8	25.0%	32.9%	0.0%	2.1%
13	17	11.8%	34.3%	0.0%	0.4%
14	6	16.7%	33.7%	0.0%	2.8%
15	15	6.7%	32.8%	0.0%	0.4%
16	6	0.0%	32.8%	0.0%	0.0%

TC: total coliform
EC: *E. coli*

Table S2. Comparison of testing and contamination rates within the study area and by flood boundary among the post-Florence and routine testing.

Data Set	Sept-Nov 2018 Post-Florence testing				Sept-Nov 2009-2017 Routine testing			
	Wells tested		% positive		Samples collected		% positive	
	#	%	TC	EC	#	%	TC	EC
**Study area**	754	0.2%	41.2%	11.7%	3,082	1.0%	35.0%	1.5%
**100-year**	335	0.2%	43.6%	15.2%	107	0.1%	31.7%	0.9%
**HAND**	161	0.4%	42.7%	11.4%	66	0.1%	22.7%	0.0%
**DFO**	160	3.0%	58.4%	20.2%	32	1.2%	46.9%	0.0%

Table S3. Comparison of testing and contamination rates within the study area and by flood boundary among post-Florence and routine testing with and without buffer.

Data Set	100 m Buffer	2018 Post-Florence				2009-2017 Routine testing			
		Wells tested		% positive		Samples Collected		% positive	
		#	%	TC	EC	#	%	TC	EC
**100-year**	No	243	0.2%	43.6%	15.2%	107	0.1%	31.7%	0.9%
	Yes	335	0.3%	44.4%	15.8%	255	0.2%	37.6%	1.2%
**HAND**	No	96	0.4%	42.7%	11.4%	66	0.1%	22.7%	0.0%
	Yes	161	0.6%	40.9%	10.5%	127	0.5%	26.8%	0.0%
**DFO**	No	79	3.0%	58.4%	20.2%	32	1.2%	46.9%	0.0%
	Yes	160	6.1%	53.1%	16.3%	99	3.8%	40.4%	2.0%

Table S4. Estimated area and number of private wells potentially impacted by Hurricane Florence inside and outside flood boundaries.

Flood boundary	Estimated area impacted		Proximity to boundary	Well estimates	
	km^2 (mi^2)	% study area		#	%
**100-year**	11,222 (4,332)	25.4%	Inside	108,397	34.7%
			Outside	203,446	65.3%
**HAND**	4,864 (1,878)	11.0%	Inside	25,621	8.2%
			Outside	286,222	91.8%
**DFO**	2,302 (888)	5.2%	Inside	2,610	0.8%
			Outside	309,233	99.2%

Table S5: Contingency table used for calculating validity metrics.

	EC Positive	EC Negative
Inside boundary	True positive (TP)	False positive (FP)
Outside boundary	False negative (FN)	True negative (TN)
Accuracy= (TP+TN)/(TP+FP+FN+TN)		
Sensitivity= TP/(TP+FN)		
Specificity=TN/(TN+FP)		

Table S6. Well water contamination rates within the study area and by flood boundary between the post-Florence and routine testing.

Flood boundary	Proximity to boundary	Post-Florence 2018		Routine 2009-2017	
		TC	EC	TC	EC
Study area	Inside	41.2%	11.7%	35.4%	1.5%
100-year	Inside	44.4%	15.8%	37.6%	1.2%
	Outside	35.7%	8.4%	32.2%	1.4%
HAND	Inside	40.9%	10.5%	26.8%	0.0%
	Outside	41.3%	12.0%	35.8%	1.6%
DFO	Inside	53.1%	16.3%	40.4%	2.0%
	Outside	38.0%	10.4%	35.2%	1.5%

Table S7. Coefficient results from the TC logistic regression

Predictor	Estimate	Std Error	z value	Pr(>|z|)
(Intercept)	-0.36	0.08	-4.73	0.00
Inside HAND boundary	-0.08	0.08	-0.90	0.37
**Inside DFO boundary**	**0.25**	**0.08**	**3.09**	**2.00E-03**
Inside FEMA 100 boundary	0.14	0.08	1.69	0.09
% BIPOC in block	0.04	0.09	0.49	0.62
% BG renting	-0.16	0.10	-1.72	0.09
% BG below poverty line	-0.13	0.10	-1.40	0.16
% BG without medical insurance	0.03	0.10	0.28	0.78
# of tests in tract	-0.31	0.35	-0.89	0.37
# of wells in tract	0.08	0.19	0.39	0.70
% of tract tested	0.25	0.29	0.87	0.39
% well users in tract	-0.13	0.11	-1.22	0.22

Bold text indicates significant variables

Table S8. Coefficient results from the EC logistic regression.

Predictor	Estimate	Std. Error	z value	Pr(>|z|)
(Intercept)	-2.19	0.13	-16.52	< 2e-16
Inside HAND boundary	-0.18	0.13	-1.37	0.17
Inside DFO boundary	0.19	0.11	1.69	0.09
**Inside FEMA 100 boundary**	**0.29**	**0.13**	**2.25**	**0.02**
% BIPOC in block	-0.20	0.17	-1.21	0.23
% BG renting	-0.15	0.14	-1.03	0.30
% BG below poverty line	-0.01	0.15	-0.08	0.94
% BG without medical insurance	0.00	0.16	-0.01	0.99
**# of tests in tract**	**-1.30**	**0.57**	**-2.28**	**0.02**
# of wells in tract	0.41	0.33	1.25	0.21
**% of tract tested**	**1.19**	**0.46**	**2.59**	**0.01**
% well users in tract	-0.21	0.16	-1.30	0.19

Bold text indicates significant variables

Table S9. Model performance for TC and EC logistic regressions.

Performance	TC model	EC model
Area under curve (AUC)	0.60	0.71
Accuracy	0.59	0.89
Precision	0.20	0.02
Recall	0.53	0.50

Table S10. Contingency table for 100-year boundary identifying EC positive and negative samples.

	EC Positive	EC Negative
Inside boundary	6.9% (52)	37.5% (283)
Outside boundary	4.8% (36)	50.8% (383)
Accuracy: 57.7%		
Sensitivity: 59.1%		
Specificity: 57.5%		

Table S11. Contingency table for HAND boundary identifying EC samples.

	EC Positive	EC Negative
Inside boundary	2.3% (17)	19.1% (144)
Outside boundary	9.4% (71)	57.8% (522)
Accuracy: 71.4%		
Sensitivity: 19.3%		
Specificity: 78.4%		

Table S12. Contingency table for DFO boundary identifying EC samples.

	EC Positive	EC Negative
Inside boundary	2.9% (26)	14.8% (134)
Outside boundary	6.9% (62)	58.9% (532)
Accuracy: 74.0%		
Sensitivity: 29.5%		
Specificity: 79.9%		

Table S13. Tract level summary statistics of flood boundary ability to identify EC positive samples based on percent of tract flooded.

Tract GEOID	# tests	# EC+ tests	100-year boundary			HAND boundary			DFO boundary		
			% tract flooded	Sensitivity	Specificity	% tract flooded	Sensitivity	Specificity	% tract flooded	Sensitivity	Specificity
37013930501	9	2	48.49%	100%	71%	22.97%	0%	100%	9.26%	50%	86%
37013930600	11	3	12.70%	33%	88%	6.61%	0%	100%	2.05%	67%	75%
37013930800	6	3	22.34%	100%	0%	22.61%	33%	67%	3.11%	0%	100%
37019020204	19	2	12.04%	100%	88%	8.35%	50%	94%	2.27%	50%	94%
37031970101	8	1	3.54%	0%	100%	0.00%	0%	100%	0.00%	0%	100%
37031970102	77	9	10.32%	100%	87%	5.70%	11%	99%	9.25%	0%	100%
37031970103	33	5	93.43%	100%	82%	0.00%	0%	100%	6.92%	20%	96%
37031970301	4	2	48.12%	100%	0%	9.71%	0%	100%	5.70%	0%	100%
37031970303	2	1	46.25%	100%	0%	0.00%	0%	100%	0.00%	0%	100%
37031970304	9	2	73.21%	100%	71%	0.00%	0%	100%	0.25%	0%	100%
37031970501	16	5	56.92%	100%	55%	93.51%	0%	55%	13.97%	40%	82%
37031970504	13	3	94.87%	100%	70%	0.00%	100%	100%	7.86%	0%	100%
37031970702	36	3	25.43%	100%	91%	27.90%	0%	91%	3.94%	100%	91%
37031970704	13	1	32.24%	100%	92%	25.99%	100%	92%	6.09%	100%	92%
37031971002	2	2	49.95%	0%	-	0.00%	100%	-	3.24%	0%	-
37047930400	9	1	37.45%	100%	88%	38.82%	0%	88%	2.81%	0%	100%
37047931300	36	4	27.98%	100%	88%	27.46%	100%	88%	10.65%	100%	88%
37051003001	2	1	3.88%	0%	100%	6.04%	100%	100%	2.46%	0%	100%
37061090701	35	12	27.74%	100%	48%	35.03%	0%	57%	24.48%	100%	48%
37079950300	1	1	10.77%	0%	-	21.57%	83%	-	6.10%	0%	-
37101041103	1	1	10.07%	0%	-	10.25%	0%	-	0.00%	0%	-
37125950100	1	1	20.71%	0%	-	37.05%	0%	-	2.92%	0%	-
37137950102	1	1	33.64%	0%	-	6.85%	0%	-	10.33%	0%	-
37137950201	5	1	4.29%	100%	75%	6.81%	0%	75%	4.16%	0%	100%
37141920203	89	7	37.56%	100%	91%	39.71%	100%	91%	9.03%	100%	91%
37141920204	27	8	63.47%	100%	58%	31.81%	100%	58%	11.30%	100%	58%
37141920300	17	1	18.80%	100%	94%	22.77%	100%	93%	4.49%	100%	94%
37141920403	16	2	34.19%	100%	86%	49.71%	100%	86%	14.71%	100%	86%
37141920501	12	1	24.12%	100%	91%	31.98%	100%	91%	4.17%	100%	91%
37141920502	10	1	68.73%	100%	89%	90.47%	100%	88%	24.82%	100%	89%
37163971000	8	1	12.18%	100%	86%	40.84%	100%	86%	9.96%	100%	81%
**Count**	528	88	-	-	-	-	-	-	-	-	-
**Average**	-	-	34.4%	75.0%	72.6%	23.0%	44.4%	88.3%	7.0%	42.8%	90.1%
**Median**	-	-	28.0%	100%	86.5%	22.6%	11.0%	91.5%	5.7%	20.0%	93.0%

Table S14. Tract level summary statistics of high flooded tracts of flood boundary ability to identify EC positive samples based on percent of tract flooded.

Tract GEOID	# tests	# EC+ tests	100-year boundary			HAND boundary		
			% tract flooded	Sensitivity	Specificity.	% tract flooded	Sensitivity	Specificity
37013930501	9	2	48.49%	100%	71%	-	-	-
37031970103	33	5	93.43%	100%	82%	-	-	-
37031970301	4	2	48.12%	100%	0%	-	-	-
37031970303	2	1	46.25%	100%	0%	-	-	-
37031970304	9	2	73.21%	100%	71%	-	-	-
37031970501	16	5	56.92%	100%	55%	93.51%	0%	55%
37031970504	13	3	94.87%	100%	70%	-	-	-
37031970702	36	3	25.43%	100%	91%	27.90%	0%	91%
37031970704	13	1	32.24%	100%	92%	25.99%	100%	92%
37031971002	2	2	49.95%	0%	-	-	-	-
37047930400	9	1	37.45%	100%	88%	38.82%	0%	88%
37047931300	36	4	27.98%	100%	88%	27.46%	100%	88%
37061090701	35	12	27.74%	100%	48%	35.03%	0%	57%
37125950100	1	1	-	-	-	37.05%	0%	-
37137950102	1	1	33.64%	0%	-	-	-	-
37141920203	89	7	37.56%	100%	91%	39.71%	100%	91%
37141920204	27	8	63.47%	100%	58%	31.81%	100%	58%
37141920403	16	2	34.19%	100%	86%	49.71%	100%	86%
37141920501	12	1	-	-	-	31.98%	100%	91%
37141920502	10	1	68.73%	100%	89%	90.47%	100%	88%
37163971000	8	1	-	-	-	40.84%	100%	86%
**Count**	381	65	18	-	-	13	-	-
**Average**	-	-	50.0%	88.9%	67.5%	43.9%	61.5%	80.9%
**Median**	-	-	47.2%	100.0%	76.5%	37.1%	100%	88.0%

Table S15. Number of samples taken within the study area and by flood boundary between the post-Florence and 2017 routine testing.

Flood boundary	Proximity to boundary	2018 Post-Florence		2017 Routine testing	
		#	%	#	%
Study area	Inside	754	0.2%	228	0.1%
100-year	Inside	335	0.3%	16	0.0%
	Outside	419	0.4%	212	0.2%
HAND	Inside	161	0.6%	10	0.0%
	Outside	593	0.2%	218	0.1%
DFO	Inside	160	6.1%	6	0.2%
	Outside	594	0.2%	222	0.1%

Table S16. Number of samples collected during the three campaigns by county.

County	2018 Post-Florence	2009-2017 Routine testing	2017 Routine testing
Beaufort	45	85	4
Bladen	8	40	6
Brunswick	51	76	7
Carteret	233	77	14
Columbus	69	325	35
Craven	0	22	2
Cumberland	2	442	45
Duplin	61	102	13
Greene	1	9	0
Harnett	4	73	10
Hoke	0	62	7
Hyde	1	11	1
Johnston	22	427	36
Jones	4	15	0
Lee	0	42	1
Lenoir	3	31	2
Moore	8	266	38
New Hanover	0	2	0
Onslow	0	35	9
Pamlico	12	49	1
Pender	204	278	22
Pitt	1	29	0
Richmond	2	56	2
Robeson	6	15	0
Sampson	9	273	57
Scotland	7	41	3
Wayne	0	85	10
Wilson	0	114	17

Table S17. County population and well using characteristics.

Name	County population	# well users	# of wells	% reliant on wells
Beaufort	47,759	32745	17,027	68.6%
Bladen	35,190	22781	10,921	64.7%
Brunswick	107,431	64847	39,325	60.4%
Carteret	66,469	39596	21,428	59.6%
Columbus	58,098	41275	18,651	71.0%
Craven	103,505	20542	9,255	19.9%
Cumberland	319,431	72927	29,591	22.8%
Duplin	58,505	34276	15,032	58.6%
Greene	21,362	1875	708	8.8%
Harnett	114,678	61143	24,502	53.3%
Hoke	46,952	28414	10,899	60.5%
Hyde	5,810	1612	994	27.8%
Johnston	168,878	124606	47,917	73.8%
Jones	10,153	2310	1,093	22.8%
Lee	57,866	21440	9,057	37.1%
Lenoir	59,495	9254	4,151	15.6%
Moore	88,247	36659	17,512	41.5%
New Hanover	202,667	56235	25,718	27.8%
Onslow	177,772	19966	8,529	11.2%
Pamlico	13,144	1685	944	12.8%
Pender	52,217	41321	19,008	79.1%
Pitt	168,148	22133	9,367	13.2%
Richmond	46,639	12865	5,594	27.6%
Robeson	134,168	42867	16,517	32.0%
Sampson	63,431	47819	20,382	75.4%
Scotland	36,157	16119	6,674	44.6%
Wayne	122,623	62010	26,483	50.6%
Wilson	81,234	26544	11,296	32.7%

Table S18. Average demographics of the well using and tested populations.

Demographic	Population	Study area	100-year	HAND	DFO
% BIPOC	Well Using	36.8%	33.2%	37.2%	37.7%
	Tested	26.7%	23.8%	27.9%	27.3%
% Poverty	Well Using	15.2%	14.9%	15.0%	16.7%
	Tested	12.8%	11.9%	13.6%	13.3%
% Renters	Well Using	24.9%	24.8%	24.6%	23.2%
	Tested	17.6%	15.7%	17.2%	13.6%
% Uninsured	Well Using	11.9%	11.9%	12.0%	12.9%
	Tested	9.3%	8.2%	9.2%	7.9%
